# Development and Validation of Narrative Competence Scale for Medical Students

**DOI:** 10.5334/pme.1707

**Published:** 2026-02-17

**Authors:** Shao-Yin Chu, Hung-Che Wang, Bang-Yuan Kuo, Meei-Ju Lin, Yu-Che Chang, Chi-Wei Lin

**Affiliations:** 1 Department of Medical Education and Paediatrics, Hualien Tzu Chi Hospital, Buddhist Tzu Chi Medical Foundation, Hualien, Taiwan; 2 Center for Innovation and Medical Education Research, Hualien Tzu Chi Hospital, Buddhist Tzu Chi Medical Foundation, Hualien, Taiwan; 3 Hualien Office, The Garden of Hope Foundation, Hualien, Taiwan; 4 Department of Emergency Medicine, Chang Gung Memorial Hospital, Linkou, Taoyuan, Taiwan; 5 Department of Counselling & Clinical Psychology, National Dong Hwa University, Hualien, Taiwan

## Abstract

**Background:**

Narrative competence in medicine enabled physicians to listen to patients’ illness experiences, construct their narratives, and enhance reflective practice, empathy, humanistic literacy, and person-centered care. However, systematic research and clear definitions for measurable indicators of narrative competence assessment were lacking. This study aimed to develop and validate a narrative competence scale for medical students.

**Methods:**

A 57-item draft scale was developed, and categorized into four dimensions and eleven sub-dimensions after interdisciplinary literature review. Three rounds of the Delphi method were conducted with eleven experts specializing in narrative medicine. Pilot testing involved 200 fifth- and sixth-year medical students in Taiwan (136 males, 64 females). Confirmatory factor analysis CFA) and Cronbach’s α were used to evaluate the NCS-MS’s reliability and validity.

**Results:**

The NCS-MS was refined through three rounds of the Delphi method, focusing on item revisions and sub-dimension definitions. The results of CFA indicated a good fit for the four-factor model (RMSEA = 0.055, SRMR = 0.045, GFI = 0.911, NFI = 0.926, IFI = 0.971, CFI = 0.970). The Cronbach’s α coefficients for the four dimensions ranged from 0.797 to 0.942, with an overall α of 0.972, demonstrating excellent internal consistency reliability.

**Conclusions:**

The NCS-MS effectively evaluated the narrative competence of medical students. From the perspective of narrative medicine, the NCS-MS served as a research or teaching assessment tool for assessing medical students’ narrative competence in narrative medicine research, instructional design, and implementation.

## Introduction

The “narrative turn” in medicine, reflecting a shift from disease-centered models toward understanding human experience through stories and meaning-making, has gained increasing attention in medical education. Grounded in the broader narrative turn in the humanities and social sciences [1], these approaches address the limitations of traditional biomedical training [2], which often underemphasizes psychosocial and sociocultural contexts in clinical decision making. Within this context, Charon [3] introduced the concept of narrative medicine, describing a form of clinical practice strengthened by narrative competence, defined as the capacity to recognize, absorb, interpret, and be moved by patients’ illness stories. Within the framework of narrative medicine, narrative competence refers to the ability of medical personnel to construct meaningful narratives of patients’ illness experiences through attentive listening, interpretation, and reflective engagement. This competence enables clinicians to understand patients’ psychosocial backgrounds, establish therapeutic relationships, and incorporate patients’ perspectives into clinical reasoning and care planning [4,5].

Narrative medicine has been increasingly adopted as a pedagogical approach in medical education, with empirical studies reporting improvements in learners’ understanding of patients’ illness experiences, broader psychosocial contexts, empathy, and communication skills [4,6]. However, evaluations of narrative medicine interventions have primarily relied on learners’ self- reported perceptions and attitudinal changes rather than direct assessments of narrative competence itself [4,7,8]. Consequently, the extent to which narrative medicine education contributes to the development of narrative competence remains insufficiently understood Addressing this issue requires a clearer articulation of the conceptual scope and core components of narrative competence within narrative medicine. To clarify these components, Chu et al. conducted in depth interviews with 15 Taiwanese healthcare professionals engaged in narrative medicine and medical humanities to explore their narrative practices and professional experiences [9]. Through thematic analysis, four core dimensions of narrative competence were identified: Narrative Horizon, Narrative Construction, Medical Relationship, and Medical Care. Narrative Horizon reflects healthcare professionals’ narrative perspectives and beliefs and represents the cognitive dimension of narrative competence. Narrative Construction includes four sub-dimensions, listening, understanding, thinking, and representation, which describe how illness narratives are formed and interpreted. Medical Relationship comprises empathy, communication, affiliation, and intersubjectivity, emphasizing relational aspects of clinical encounters. Medical Care encompasses responsive care, balancing act, and medical reflection, capturing the integration of narrative understanding into clinical practice. Together, these dimensions provided the conceptual foundation for developing a scale to assess narrative competence among medical personnel, which constitutes the primary aim of the present study.

In addition to this empirically derived framework, the development of the narrative competence scale was further informed by interdisciplinary perspectives from literature, psychology, and philosophy, which helped refine the conceptual meaning of narrative competence for item construction [2]. From the perspective of literary studies, narrative competence involves attending to and comprehending narrative elements within patients’ stories, including plot, character, metaphor, symbolism, and temporality [10,11,12]. From a psychological perspective, narrative competence entails recognizing underlying emotions, motivations, intentions, and actions embedded in illness narratives and responding appropriately to patients’ emotional expressions [13,14]. From a philosophical perspective, particularly influenced by Lévinas’ philosophy of the Other, narrative competence emphasizes ethical attentiveness, intersubjective engagement, and reflection on patients as narrative subjects rather than solely as clinical cases [17,18,19,20]. These interdisciplinary perspectives collectively informed the operationalization of narrative competence into measurable dimensions and items.

According to Bandura [21], individual performance is shaped by the interaction between internal psychological factors, such as self-efficacy, cognitive strategies, emotional responses, and observable behaviors. From this perspective, competence can be understood as a multidimensional construct that encompasses both internal processes and external expressions. In educational research, self-report questionnaires are commonly used to assess internal cognitive and affective processes that cannot be directly observed, as they allow respondents to report their perceptions, reflections, and experiences in a structured manner [22,23]. Narrative competence comprises core components such as empathy, reflection, interpretation, and responsiveness to patients’ stories, as described by Charon [2] and Chu et al. [9]. These components involve cognitive and emotional processes that are not readily captured through behavioral observation alone. Accordingly, self-report measures represent an appropriate methodological approach for assessing these aspects of narrative competence, as they provide a systematic means of examining constructs that include internal psychological dimensions, while complementing information obtained from observational approaches.

Despite the increasing integration of narrative medicine into medical education, assessments of narrative competence have largely relied on reflective writing or self-perceived outcomes, with limited use of standardized instruments [24]. Within competency-based medical education, validated assessment tools are essential for identifying learners’ developmental needs and evaluating curricular effectiveness [25]. This study proposes that an inquiry into the narrative competence of medical personnel necessitates a preliminary understanding of the scope and depth of narrative competence. Following this, it becomes imperative to operationalize the concept of narrative competence into tangible, quantifiable assessment criteria. Thus, this study aimed to develop the narrative competence scale and test its reliability and validity.

## Methods

**Figure 1:**
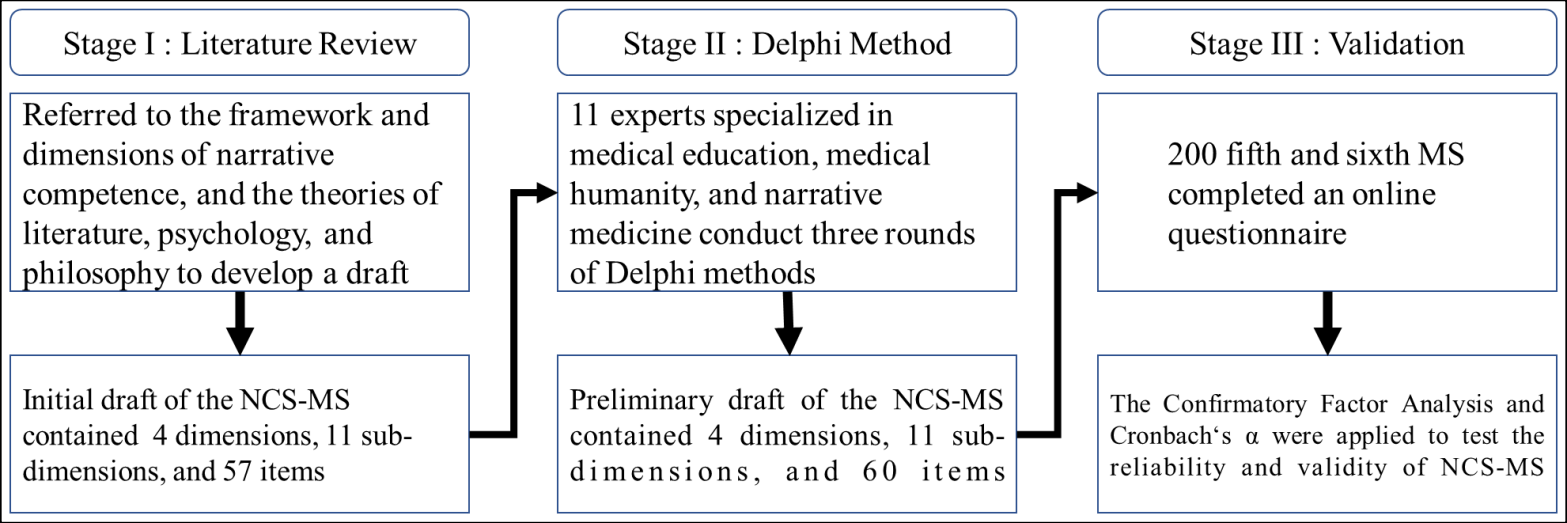
Stages of study.

This study adopted a three-stage design for the development and validation of the Narrative Competence Scale – Medical Students (NCS-MS) (Figure 1). Stage 1 involved an interdisciplinary literature review to inform the conceptual framework and item generation; Stage 2 applied a modified Delphi method to refine items and establish content validity; and Stage 3 comprised a pilot study to examine the psychometric properties of the NCS-MS. Approval was obtained from the Institutional Review Board of the Buddhist Tzu Chi General Hospital (IRB 110-034-B).

### Stage I: Interdisciplinary literature Review

The major conceptual framework and definitions of narrative competence for this study derived from narrative medicine and related interdisciplinary scholarship. The framework proposed by Chu et al. [9], grounded in narrative medicine and informed by interviews with healthcare and medical education professionals, was included as one reference reflecting the cultural and clinical context of medical education. Additional theoretical perspectives from literature studies [10,11,12], psychology [13,14,15,16], and philosophy and aesthetics [17,18,19,20] were incorporated to further refine the conceptualization of the construct. Guided by this integrated conceptual foundation, an initial version of the Narrative Competence Scale-Medical Students (NCS-MS) was developed, comprising four primary dimensions, eleven sub-dimensions, and 57 items.

### Stage II: The Delphi Method

Content validity was determined to measure the accuracy with which the items reflected the scale’s construct. This stage of the study employed three rounds of the Delphi method for content validation, involving experts in medical education, medical humanities, and narrative medicine, each with more than five years of experience as a medical teacher. In Round One, a cover letter, consent form, and a set of questions via Google Sheets were emailed to a panel of 11 experts. The cover letter included information on the reasons for the experts’ selection, the background of the research, and instructions on how to rate the survey items. Participants were requested to rate each item on a 5-point Likert scale based on its importance to the construct (5 = extremely important to 1 = not important). Additionally, panellists were encouraged to add comments in the provided comment box and suggest additional components through an open-ended question at the end of the questionnaire. In Rounds Two and Three, a modified version of the NCS-MS and a rating scale like that in Round One were sent to the experts by email to continue rating the importance of the NCS-MS’s construct. The Delphi method was concluded upon achieving consensus after completing round three and establishing a preliminary NCS-MS which contained four primary dimensions, eleven sub-dimensions, and 60 items.

Quantitative data in three rounds were analysed using the Statistical Package for Social Sciences (SPSS) version 18. The predetermined criteria for evaluating the responses were a mean score > 4, standard deviation < 1, mode > 4, and interquartile range < 0.6 [27,28,29]. Content validity was assessed using item-level (I-CVI) and scale-level indices (S-CVI/Ave and S-CVI/UA) based on expert ratings from the final Delphi round, with acceptable thresholds defined as I-CVI ≥ .78, S-CVI/Ave ≥ .90, and S-CVI/UA ≥ .80 [30,31].

### Stage III: Validation

To examine the validity and reliability of the NCS-MS, the scale was administered online to medical students recruited through social network groups. A total of 200 fifth- and sixth-year medical students (136 male and 64 female; 104 fifth-year and 96 sixth-year) from twelve medical universities in Taiwan completed the survey. Responses were recorded using a six-point Likert scale ranging from 1 (Completely disagree) to 6 (Completely agree). The sample size met established recommendations for confirmatory factor analysis in scale development, which suggest that 150 to 200 participants are sufficient when factor loadings are moderate to strong and the construct is theoretically grounded [32,33]. Thus, the final sample of 200 students was adequate for the planned analyses. All participants were enrolled in a six-year medical curriculum and had entered clinical rotations by their fifth and sixth years, providing direct patient care experience. This clinical exposure rendered them appropriate respondents for the NCS-MS, which assesses competencies developed through clinical engagement and narrative-based communication.

Confirmatory Factor Analysis (CFA) and Cronbach’s α in the validation stage were employed to establish the instrument’s construct validity, convergent validity, discriminant validity, and internal consistency reliability [26]. Since the NCS-MS was developed based on the theoretical framework of narrative competence from the works of Chu et al. [9] and underwent three rounds of refinement and validation through the Delphi expert consensus method, its structure was supported by a robust theoretical foundation and content consistency. This stage employed confirmatory factor analysis (CFA) to evaluate the goodness of fit between the theoretical model of the NCS-MS and the empirical data, ensuring that the variables align with the expected construct structure. Unlike exploratory factor analysis (EFA), typically used to identify underlying factor structures without prior assumptions, CFA was applied when testing models guided by predefined theoretical frameworks [34]. As Kline [32] noted, CFA is preferred when a study has a clear theoretical perspective and conceptual framework, thus eliminating the need for preliminary EFA. An initial CFA at the item level was performed to examine factor loadings, residuals, and the unidimensionality of items within each sub-dimension. Establishing item-level unidimensionality ensured that item parceling was introduced only after adequate psychometric performance had been confirmed. Based on these results, item parceling was subsequently applied as a secondary model refinement procedure for the Narrative Construction, Medical Relationship, and Medical Care sub-dimensions, in accordance with methodological recommendations for multidimensional constructs [35,36]. CFA was conducted using the maximum likelihood estimation method in three steps. First, the factor loadings of each item were examined, with a factor loading of less than 0.5 set as the criterion for item deletion. Second, the goodness of fit of the NCS-MS theoretical model was evaluated using various fit indices: Chi-Square (χ^2^), Root Mean Squared Error of Approximation (*RMSEA*), Standardized Root Mean Square Residual (*SRMR*), Goodness of Fit Index (*GFI*), Normative Fit Index (*NFI*), Relative Fit Index (*RFI*), Incremental Fit Index (*IFI*), Comparative Fit Index (*CFI*), Parsimony Normed Fit Index (*PNFI*), Parsimony Comparative Fit Index (*PCFI*), and Parsimony Goodness of Fit Index (*PGFI*) [37]. Finally, the construct validity of the NCS-MS was assessed by evaluating its convergent and discriminant validity. Convergent validity was tested using Component Reliability (*CR*) and Average Variance Extracted (*AVE*), while discriminant validity was tested using the square root of the average variance [37]. Additionally, because the NCS-MS had 60 observed variables, ‘item parceling’ was used for the Narrative Construction, Medical Relationship, and Medical Care sub-dimensions to minimize the risk of underestimating model fit in CFA [35,36].

After confirming the factor structure and model fit through CFA to establish construct validity, convergent validity, and discriminant validity, internal consistency reliability was assessed using both Cronbach’s α and composite reliability (CR), which corresponds to Raykov’s reliability coefficient (ρ) derived from CFA factor loadings [38]. CFA was conducted using AMOS software version 21, and Cronbach’s α was calculated using SPSS version 18.

## Results

### The Delphi Method

The Delphi process was conducted in three rounds. In the first round, all 57 items received mean importance ratings above 4.0, with most items showing a mode of 5 and low variability, indicating high initial consensus. Item-level content validity indices ranged from .73 to 1.00, with scale-level indices of S-CVI/UA = .72 and S-CVI/Ave = .89. Expert feedback primarily concerned wording, conceptual clarity, and item appropriateness. Based on this feedback, 47 items were revised, four new items were added, and two redundant items were deleted. In the second round, expert consensus remained high, with all items again exceeding a mean rating of 4.0 and showing low dispersion. Revisions focused on further wording refinement, clarification of conceptual boundaries, and reduction of respondent burden. Consequently, 40 items were revised, two items were deleted, and one previously removed item was reinstated based on expert judgment. The third round demonstrated stable and complete consensus. All items achieved an I-CVI of 1.00, and both S-CVI/Ave and S-CVI/UA reached 1.00, exceeding established criteria for adequate content validity. Following the three-round Delphi process, the NCS-MS was finalized with four dimensions, eleven sub-dimensions, and a total of 60 items.

### Confirmatory factor analysis

**Figure 2:**
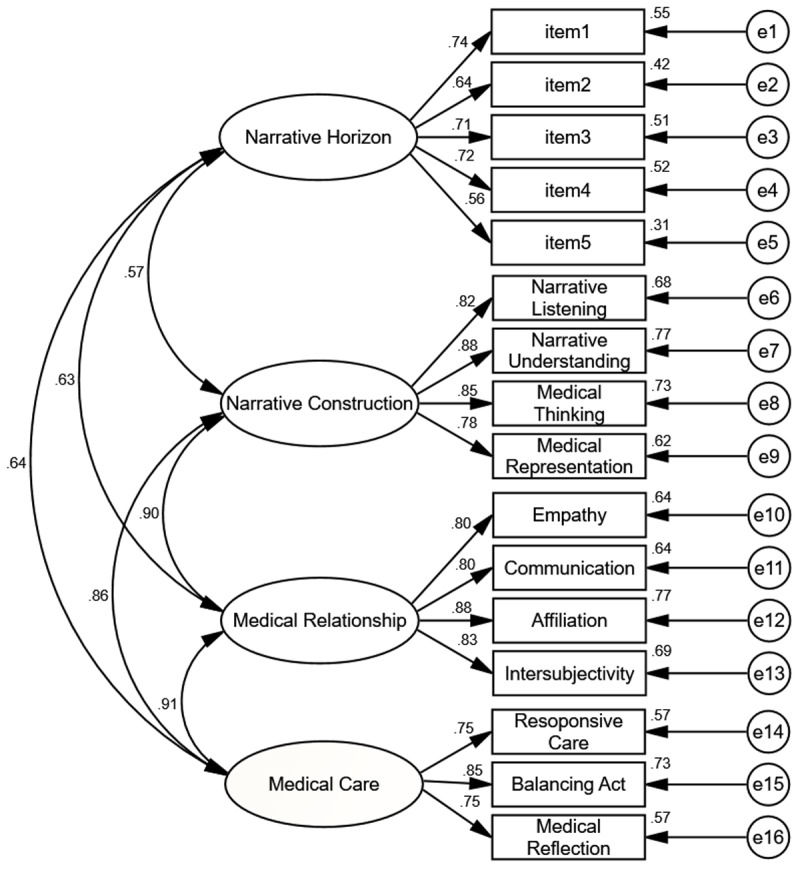
Fitted path diagram of NCS-MS.

After the Delphi process, this study validated the four-factor oblique model using CFA. Initially, it was found that the factor loading of item 6 in the narrative horizon dimension was 0.36, which is below the standard threshold of 0.50 [39], leading to its deletion. Subsequently, CFA was conducted on the remaining 59 items using maximum likelihood factor analysis with oblique rotation (Figure 2). The results showed that the χ^2^ value (χ^2^ = 157.722, *p* < .05)was less than the requisite minimum threshold; however, the model exhibited a good fit according to other indices; (*RMSEA* = 0.055, *SRMR* = 0.045, *GFI* = 0.911, *NFI* = 0.926, *RFI* = 0.910, *IFI* = 0.971, *CFI* = 0.970, *PGFI* = 0.657, *PNFI* = 0.757, *PCFI* = 0.793) [32,39,40]. Therefore, the four-factor model was found to be interpretable.

Table 1 presents the results of the convergent and discriminant validity of the NCS-MS. The AVE of each dimension ranged from 0.497 to 0.698, exceeding the threshold of 0.5, and the CR of each dimension ranged from 0.798 to 0.902, surpassing the threshold of 0.6, indicating strong convergent validity of the scale. Additionally, the square root of the average variance extracted for each construct was from 0.705 to 0.830, higher than the correlation coefficients of other dimensions, suggesting good discriminant validity [37]. These results collectively support the construct validity of the NCS-MS, as they demonstrate that the items within each dimension are highly correlated (convergent validity) and that the dimensions are distinct from each other (discriminant validity) [39].

**Table 1:** Convergent and Discriminant Validity of the NCS-MS.


DIMENSION	CONVERGENT VALIDITY		DISCRIMINANT VALIDITY			
	
	CR	AVE	1	2	3	4

1. Narrative horizon	0.798	0.497	(0.705)			

2. Narrative construction	0.902	0.698	0.511	(0.830)		

3. Medical relationship	0.897	0.685	0.544	0.816	(0.827)	

4. Medical care	0.831	0.621	0.527	0.755	0.783	(0.788)


Note: In the discriminant validity table, the values in the bracket are the square root of the AVE for each construct, while the other values are the correlation coefficients between constructs.

### Internal Consistency Reliability

Internal consistency reliability of the NCS-MS was evaluated using Cronbach’s α and composite reliability (CR). At the dimension level, Cronbach’s α ranged from .797 to .942 across the four dimensions, with corresponding CR values ranging from .798 to .902 (see Table 1), indicating satisfactory reliability. At the sub-dimension level, Cronbach’s α values ranged from .802 to .889 across the Narrative Construction, Medical Relationship, and Medical Care sub-dimensions, further supporting internal consistency. The overall Cronbach’s α for the full scale was .972, indicating excellent reliability. Taken together, the Cronbach’s α and CR estimates provide strong evidence for the internal consistency reliability of the NCS-MS under both tau-equivalent and congeneric measurement assumptions.

## Discussion

Prior research has yet to establish a standardized assessment tool for evaluating narrative competence among medical personnel. Addressing this gap, the present study describes the development of the NCS-MS and provides empirical evidence supporting its validity and reliability. The consensus achieved by the Delphi expert panel provides support for the content validity of the NCS-MS, indicating that the scale items adequately reflect the conceptual domain of narrative competence. The favorable model fit indices obtained through confirmatory factor analysis suggest that the proposed four-dimension structure represents a coherent and theoretically grounded configuration of narrative competence. The satisfactory convergent and discriminant validity further indicate that the dimensions capture related yet distinguishable aspects of the construct, consistent with the underlying conceptual framework. Although the chi-square statistic remained significant, this result is likely attributable to sample size sensitivity rather than substantive model misspecification. In addition, the acceptable internal consistency observed across the overall scale, its four dimensions, and eleven sub-dimensions supports the internal coherence of the measurement structure. Taken together, these findings provide empirical support for the narrative competence framework proposed by Chu et al. [9] and demonstrate its suitability for operationalization in a standardized assessment format. Accordingly, the NCS-MS may be considered a comprehensive instrument for assessing medical students’ narrative competence across multiple dimensions (Appendix 1).

Because the validation sample consisted of fifth- and sixth-year medical students with clinical clerkship experience, the present findings primarily reflect learners who have already been exposed to patient encounters. As narrative competence involves interpreting patients’ stories and their illness experiences within clinical contexts, future applications of the NCS-MS should consider whether respondents possess sufficient clinical experience to meaningfully engage with the items. Additional validation among preclinical students, postgraduate trainees, and other healthcare professionals would clarify the stages of training at which the instrument is most appropriately used.

The NCS-MS was designed to bridge narrative medicine theory and clinical practice by grounding item development in an empirically derived narrative competence framework [9] and integrating insights from literature, psychology, and philosophy. This multidisciplinary approach enables a systematic assessment of students’ abilities to identify key narrative elements, interpret patients’ emotions and intentions, and adopt a patient-centered and reflective stance in clinical contexts. The NCS-MS employs a structured self-report format that enables students to evaluate their narrative competence in a standardized and quantifiable manner. Although self-report data inherently contain subjective elements, the scale’s psychometrically validated scoring structure systematically transforms these internal judgments into measurable indicators. In this way, the NCS-MS provides quantifiable information about learners’ cognitive and affective components of narrative competence while complementing observational and performance-based assessments. By operationalizing a conceptually rich construct into measurable dimensions, the NCS-MS provides an evidence-based foundation for competency-based assessment in medical education. Dimension- and sub-dimension-level profiles may be used to identify learners’ strengths and areas requiring further development, supporting targeted instructional design and structured feedback. Beyond summative assessment, the NCS-MS also holds formative value, enabling individualized reflective feedback, narrative-based coaching, and curriculum-level evaluation. The NCS-MS provides standardized quantitative data for evaluating narrative competence. Beyond individual assessment, aggregated scale results may support curriculum evaluation by allowing educators to examine cohort-level performance patterns, compare students’ learning outcomes before and after course participation, and identify areas requiring additional instructional reinforcement. Accordingly, the NCS-MS functions not only as a learner-centered assessment tool but also as a program-level indicator that can inform curricular refinement and monitor overall trends in narrative competence development.

Previous research indicates that narrative medicine interventions can enhance empathy, narrative understanding, and shared decision-making [3], yet validated instruments for assessing narrative competence remain limited [4,8]. This study provides initial psychometric evidence for the NCS-MS as a measure of narrative competence among medical students. However, the findings should be interpreted cautiously, as the study did not examine educational effects, longitudinal development, or broad generalizability. Future research should replicate the validity and factor structure of the NCS-MS across institutions, learner groups, and cultural contexts, using longitudinal and multi-center designs to further evaluate its stability and educational utility.

## Limitation

This study has several limitations. First, the conceptual framework guiding item generation was informed by a qualitative study conducted within a specific educational and cultural context. Although the NCS-MS items were developed through interdisciplinary theoretical integration rather than direct adaptation of qualitative data, the stability of the framework and factor structure requires further examination across diverse cultural, institutional, and clinical settings.

Second, the validation sample consisted of fifth- and sixth-year medical students from medical schools in Taiwan. While this sample size was adequate for the planned psychometric analyses, the sampling frame may limit the generalizability of the findings to learners at other stages of training or to healthcare professionals from different disciplines. Future studies should validate the NCS-MS among preclinical students, postgraduate trainees, and other healthcare professionals, such as nurses and allied health practitioners, to establish its broader applicability.

Third, this study relied solely on self-report data, which may limit external validity. Although self-report measures are appropriate for assessing reflective, cognitive, and affective components of narrative competence, observable narrative behaviors in clinical encounters were not assessed. Future research should triangulate self-report data with performance-based evidence, such as narrative essays, OSCE communication assessments, or patient feedback, to strengthen the validity argument for the NCS-MS.

Fourth, the NCS-MS comprises 59 items across four dimensions and eleven sub-dimensions, which may impose response burden in time-constrained educational settings. Future research may consider developing and validating a short-form version of the NCS-MS to enhance feasibility while retaining adequate psychometric properties.

Finally, the NCS-MS was developed and validated in Chinese within the Taiwanese cultural context. Although its design considered conceptual transferability, careful translation and cross-cultural adaptation are required before application in other linguistic and cultural settings. Replication and cross-cultural validation studies are therefore necessary to examine the applicability and robustness of the NCS-MS across international contexts.

## Conclusion

This study is anchored in the empirical framework of narrative competence, weaving together elements from literature, psychology, and philosophy to form a cohesive foundation. It identified four dimensions and eleven sub-dimensions, which served as the basis for developing a scale. The scale comprises carefully designed items that reflect medical students’ subjective perceptions and self-assessments of their narrative competence, providing a detailed lens through which to understand their internal experiences and reflections in this critical domain. The findings of this study support the NCS-MS as a valid and reliable instrument for assessing narrative competence among medical students. Implementation of this instrument in narrative medicine programmes can help medical teachers understand the narrative competence of students and design adequate narrative curricula and instructional strategies to enhance narrative competence of students.

## Appendix

**Appendix 1:** The dimensions, sub-dismensions, and items of the formal NCS.

**Dimension 1: Narrative Horizon**

**1.** I believe that medical care should be patient-centric and that clinicians should treat their patients as unique individuals.

**2.** I believe healthcare professionals should be able to derive meaning and insight from patients’ narratives.

**3.** I believe medical considerations should extend beyond merely eliminating or alleviating symptoms to include the pursuit of overall well-being.

**4.** I think that medical care should focus on not only ‘curing’ but also ‘healing’ the mind, body, and spirit.

**5.** I believe healthcare professionals should periodically reflect on the limitations of empirical biomedicine.

**Dimension 2: Narrative Construction**

**Subdimension 2.1: Narrative listening**

**6.** I can maintain attention and focus on the narratives of patients and their family members.

**7.** I approach the emotions and thoughts of patients and their family members with a nonjudgemental attitude.

**8.** I can identify key pieces of information in the narratives of patients and their family members.

**9.** I observe and understand the body language of patients and their family members.

**10.** I listen to the narratives of patients and their family members with openness and curiosity.

**11.** I understand the relationship between patients’ illnesses and their real-life contexts.

**12.** When confronted with vague or inconsistent narratives from patients and their family members, I can still accept and respond appropriately.

**Subdimension 2.2: Narrative understanding**

**13.** I can understand patients’ perspectives and interpretations of their illnesses.

**14.** I can grasp how major life history events affect patients.

**15.** I can comprehend the patients’ narratives, including key elements such as plots, characters, and metaphors.

**16.** I am attentive to the psychological, social, and cultural contexts of patients’ illnesses.

**17.** I can understand patients’ descriptions of their illness journeys, such as living with the disease and recovery.

**18.** Beyond patients’ functional limitations, I can recognise their potential for recovery and strengths.

**19.** I can understand where patients are experiencing discomfort.

**Subdimension 2.3: Narrative thinking**

**20.** I can reflect on the meaning of authentic narratives presented in medical contexts.

**21.** I can understand the meaning of illness in a patient’s life.

**22.** I can identify complex themes and contextual situations within patients’ narratives.

**23.** I can engage in further inquiry about the initial narratives of patients and their family members.

**Subdimension 2.4: Narrative representation**

**24.** I can vividly depict significant details in a patient’s narrative.

**25.** I can use my imagination to reconstruct the patient’s narrative.

**26.** I can empathise with the patient and reinterpret their narrative of their illness together with them.

**27.** I can, together with patients, holistically link the illness to their real-life situations or experiences.

**Dimension 3: Medical Relationship**

**Subdimension 3.1: Empathy**

**28.** I can understand the thoughts and feelings expressed by patients and their family members.

**29.** I empathise appropriately, considering the perspectives of patients and their family members.

**30.** When I listen to or witness a patient’s narrative, I am moved.

**31.** I can empathise with patients’ experiences and feelings related to their illness.

**32.** Even if I cannot fully understand a patient’s agony, I still strive to make them feel supported.

**Subdimension 3.2: Communication**

**33.** I encourage patients to share their illness-related life experiences.

**34.** When communicating with patients and their family members, I can express myself clearly and completely.

**35.** When communicating with patients and their family members, I promptly respond to their needs and provide feedback.

**36.** I maintain effective patient–physician interaction through dialogue and the exchange of experiences.

**37.** I actively engage in interdisciplinary collaboration and communication.

**Subdimension 3.3: Affiliation**

**38.** I can establish a cooperative relationship with patients and their family members through mutual understanding.

**39.** In my medical practice, I facilitate the learning and growth of patients and their family members while also recognising my learning and growth through them.

**40.** I understand that a patient’s narrative is a means to connect with healthcare professionals.

**41.** I perform medical tasks entrusted to me by patients with gratitude.

**42.** I can share in the illness and agony–related experiences of patients and their family members through empathetic responses.

**Subdimension 3.4: Intersubjectivity**

**43.** I can develop a contextual understanding of illness experiences together with patients, starting from their initial narratives of the disease.

**44.** I can transcend differences between myself and others (e.g. patients, family members, and peers) to understand their experiences.

**45.** I can understand and reflect on the different values in patient–physician interactions and achieve an appropriate balance.

**46.** I respect different medical professions and function within interdisciplinary teams.

**Dimension 4: Medical Care**

**Subdimension 4.1: Responsive care**

**47.** I invite patients to join my medical decision–making team and provide them with ample opportunities to express their preferred approaches to care.

**48.** I actively listen to and respond to patients’ medical opinions, including suggestions that may be challenging to adopt.

**49.** I actively respond to patients’ needs, offering personalised care.

**50.** By participating in discussions with medical peers, I aim to address my blind spots in the patient–physician relationship, thereby enhancing my communication skills with patients.

**Subdimension 4.2: Balanced act**

**51.** During the healthcare process, I address my patients’ psychological needs.

**52.** During the healthcare process, I consider the physical and spiritual aspects of diseases from a holistic perspective.

**53.** During the healthcare process, I contemplate diseases and physical, social, and spiritual aspects from a holistic perspective.

**Subdimension 4.3: Medical reflection**

**54.** In clinical care, I periodically review my physical and mental workload.

**55.** I fully understand the uncertainty/variability of clinical work and adapt myself accordingly.

**56.** I am aware of the effect of the stigmatisation of certain diseases on healthcare.

**57.** Confronted with the complexity and variability of clinical settings, I reflect on my medical actions to enhance healthcare efficacy.

**58.** I continuously reflect on my experiences in caring for patients, thereby deriving new insights and skills.

**59.** Through reflection, I attempt to perceive the meanings embedded in the disease process as opportunities for change, facilitating healing for both myself and my patient.
